# CaMKII Modulates Diacylglycerol Lipase-α Activity in the Rat Nucleus Accumbens after Incubation of Cocaine Craving

**DOI:** 10.1523/ENEURO.0220-21.2021

**Published:** 2021-10-08

**Authors:** Conor H. Murray, Andrew D. Gaulden, Alex B. Kawa, Mike Milovanovic, Aaron J. Caccamise, Jonathan R. Funke, Sachin Patel, Marina E. Wolf

**Affiliations:** 1 Department of Neuroscience, Chicago Medical School, Rosalind Franklin University of Medicine and Science, North Chicago, Illinois 60064-3095; 2 Department of Behavioral Neuroscience, Oregon Health & Science University, Portland, Oregon 97239-3098; 3 Department of Psychiatry, Vanderbilt University, Nashville, Tennessee 37240

**Keywords:** CaMKII, cocaine, diacylglycerol lipase, incubation of craving

## Abstract

Relapse is a major challenge to the treatment of substance use disorders. A progressive increase in cue-induced drug craving, termed incubation of craving, is observed after withdrawal from multiple drugs of abuse in humans and rodents. Incubation of cocaine craving involves the strengthening of excitatory synapses onto nucleus accumbens (NAc) medium spiny neurons via postsynaptic accumulation of high-conductance Ca^2+^-permeable AMPA receptors. This enhances reactivity to drug-associated cues and is required for the expression of incubation. Additionally, incubation of cocaine craving is associated with loss of the synaptic depression normally triggered by stimulation of metabotropic glutamate receptor 5 (mGlu5), leading to endocannabinoid production, and expressed presynaptically via cannabinoid receptor 1 activation. Previous studies have found alterations in mGlu5 and Homer proteins associated with the loss of this synaptic depression. Here we conducted coimmunoprecipitation studies to investigate associations of diacylglycerol lipase-α (DGL), which catalyzes formation of the endocannabinoid 2-arachidonylglycerol (2-AG), with mGlu5 and Homer proteins. Although these interactions were unchanged in the NAc core at incubation-relevant withdrawal times, the association of DGL with total and phosphorylated Ca^2+^/calmodulin-dependent protein kinase IIα (CaMKIIα) and CaMKIIβ was increased. This would be predicted, based on other studies, to inhibit DGL activity and therefore 2-AG production. This was confirmed by measuring DGL enzymatic activity. However, the magnitude of DGL inhibition did not correlate with the magnitude of incubation of craving for individual rats. These results suggest that CaMKII contributes to the loss of mGlu5-dependent synaptic depression after incubation, but the functional significance of this loss remains unclear.

## Significance Statement

Cue-induced drug craving progressively intensifies or incubates after withdrawal from extended-access drug self-administration, augmenting relapse vulnerability. Incubation of cocaine craving in rats is accompanied by robust plasticity in the NAc, including strengthening of excitatory synapses via postsynaptic AMPAR plasticity and the loss of endocannabinoid-dependent synaptic depression. Our results identify a novel mechanism that may account for this loss of synaptic depression, namely reduced activity of DGL, the enzyme that produces the endocannabinoid 2-AG, along with increased physical association of this enzyme with CaMKII, an association predicted to reduce the enzymatic activity of DGL. These findings expand our understanding of mechanisms underlying cocaine-induced alterations in endocannabinoid-dependent synaptic depression.

## Introduction

Relapse is often triggered by exposure to drug-associated cues even after prolonged abstinence (O’Brien, 2005). During abstinence, cue-induced craving intensifies over time, a phenomenon termed incubation of craving. Incubation of cocaine craving has been well documented in rats (Pickens et al., 2011) and also studied in humans (Parvaz et al., 2016), where cue-induced craving peaks between 1 and 6 months before declining by 1 year of abstinence. This trajectory is similar in rats, where craving is maximal after ∼1 month, remains high for months, and then declines, although it remains significantly elevated, compared with withdrawal day 1 (WD1), after 180 d (Lu et al., 2004).

Incubation involves adaptations in regions related to motivation and reward including the nucleus accumbens (NAc; Li et al., 2015; Wolf, 2016; Wright and Dong, 2020). The NAc serves as an interface between cortical and limbic inputs and motor-related outputs, thereby contributing to the execution of motivated behaviors (Meredith et al., 2008; Sesack and Grace, 2010). Medium spiny neurons (MSNs), the principal cell type and projection neurons of the NAc, receive glutamatergic projections from cortical and limbic regions. Incubation of cocaine craving is associated with the incorporation of high-conductance calcium-permeable AMPA receptors (CP-AMPARs) into excitatory synapses of NAc core and shell MSNs (Wolf, 2016). In the core, this begins after ∼1 month of withdrawal (Wolf and Tseng, 2012). Once present, CP-AMPAR activation is required for the expression of incubation (Conrad et al., 2008; Lee et al., 2013; Loweth et al., 2014; Ma et al., 2014). It is theoretically possible that the expression of incubation also involves a reduction in mechanisms that normally constrain excitatory transmission in the NAc, such as long-term depression (LTD).

A well characterized form of LTD in the NAc is triggered by metabotropic glutamate receptor 5 (mGlu5), leading to the generation of endocannabinoids (eCBs) that act as retrograde messengers to stimulate presynaptic cannabinoid receptor 1 (CB1R), resulting in the reduction of glutamate release from excitatory afferents in the NAc (Robbe et al., 2001, 2002; Zlebnik and Cheer, 2016; Araque et al., 2017; Augustin and Lovinger, 2018). The best characterized eCB ligands are anandamide (AEA) and 2-arachidonylglycerol (2-AG) (Ohno-Shosaku and Kano, 2014). Although some evidence implicates AEA in mediating eCB-LTD in dorsal striatum (Ronesi et al., 2004; Ade and Lovinger, 2007) and both presynaptic CB1R- and postsynaptic TRPV1 (transient receptor potential cation channel subfamily V member 1)-dependent LTD in the NAc (Grueter et al., 2010), we focused on 2-AG because its contribution to eCB-LTD in the NAc is linked to the regulation of motivated behavior (Novak et al., 2010; Bilbao et al., 2020; Folkes et al., 2020). Formation of 2-AG at excitatory synapses in the NAc is mediated by the 2-AG signalosome—a postsynaptic multiprotein complex composed of mGlu5, enzymes involved in 2-AG production, and Homer scaffolding proteins (Piomelli, 2014). Stimulation of mGlu5 activates phospholipase-C, which cleaves plasma membrane PIP2 (phosphatidylinositol 4,5-bisphosphate) to form 1,2-diacylglycerol (DAG), a second messenger that is hydrolyzed by DAG lipase-α (DGL) to yield 2-AG, which acts on presynaptic CB1Rs (Jung et al., 2007).

NAc synaptic depression, evoked electrically or via pharmacological activation of mGlu5, is abolished 1 d after acute noncontingent cocaine (Fourgeaud et al., 2004; Grueter et al., 2010), 14 d after repeated noncontingent cocaine injections (Huang et al., 2011), and after >35 d of withdrawal from extended-access cocaine self-administration (McCutcheon et al., 2011; Scheyer et al., 2014). Neither acute cocaine administration (Fourgeaud et al., 2004) nor incubation of craving (McCutcheon et al., 2011) impairs CB1R function, implying a postsynaptic locus. Indeed, alterations in the 2-AG signalosome have been observed after regimens leading to the loss of this synaptic depression, namely a reduction in mGlu5 surface expression (Fourgeaud et al., 2004; Huang et al., 2011; Loweth et al., 2014) and altered Homer levels (Fourgeaud et al., 2004) or Homer-mGlu5 associations (Loweth et al., 2014). However, no studies have assessed the enzymatic activity of DGL or its associations within the 2-AG signalosome after cocaine exposure.

Here, we used coimmunoprecipitation (co-IP) to assess associations between DGL and other components of the signalosome and also assayed enzymatic activity of DGL in the NAc core. Compared with saline controls, associations between total and phosphorylated Ca^2+^/calmodulin-dependent protein kinase II (CaMKII) and DGL were increased at incubation-relevant withdrawal times. This was accompanied by decreased DGL activity, as predicted by studies demonstrating that CaMKII phosphorylation reduces DGL activity (Shonesy et al., 2013; Park et al., 2017). However, our results fall short of demonstrating that loss of this synaptic depression contributes to incubated craving. In fact, other findings argue that this synaptic depression promotes reward seeking (Novak et al., 2010; Bilbao et al., 2020; Mitra et al., 2021).

## Materials and Methods

### Subjects and surgery

All animal procedures were performed in accordance with the regulations of the animal care committees of Rosalind Franklin University and the Oregon Health & Science University, and with the National Institutes of Health (NIH) *Guide for the Care and Use of Laboratory Animals*. Male Sprague Dawley rats (Envigo) weighing 275–300 g were housed three to a cage under a reverse 12 h light/dark cycle with food and water available *ad libitum*. One week after arrival, rats were implanted with a jugular catheter under ketamine/xylazine anesthesia (80/10 mg/kg, i.p.). Before surgery, rats received an injection of the analgesic Banamine (flunixin meglumine, 2.5 mg/kg, s.c.) to minimize postoperative discomfort. Thereafter, rats were single housed for 7 d before beginning self-administration training. Catheters were flushed daily with cefazolin (100 mg/ml in sterile 0.9% saline; 0.15 ml) during this period to prevent infection and maintain catheter patency.

### Drug self-administration and cue-induced seeking tests

Cocaine was obtained from the National Institute on Drug Abuse and dissolved in 0.9% saline. Rats self-administered cocaine (0.5 mg/kg/infusion in a 100 μl/kg volume over 3 s) or saline, during 10 consecutive sessions (6 h/d starting at zeitgeber time 15), in a chamber equipped with two nose-poke holes. Active hole nose pokes resulted in intravenous delivery of the drug paired with a 20 s light cue (white light illuminating the active hole) on a fixed ratio 1 schedule. Each infusion was followed by a 20 s time-out period. Nose poking in the inactive hole had no consequences. After 10 d of training, rats underwent forced abstinence in home cages and were handled weekly until being used for experiments on or after WD40. This regimen reliably results in incubation of cocaine craving that is robustly expressed during this withdrawal period (Conrad et al., 2008; Loweth et al., 2014). Therefore, we did not assess incubation of craving in rats used in some of our biochemical studies. However, two cohorts of rats, used for DGL activity assays, underwent cue-induced seeking tests after the same cocaine self-administration regimen to verify incubation of craving. During these seeking tests, responses in the previously active hole delivered the light cue but no drug. Responding under these conditions is our operational measure of cue-induced cocaine craving. One cohort underwent seeking tests on WD1 and WD40, and the other on WD1 and WD42.

### Coimmunoprecipitation

Rats were decapitated and bilateral NAc core was rapidly dissected from two 1 mm slices prepared with a brain matrix (ASI Instruments) using a 1.5 mm biopsy punch (Thermo Fisher Scientific). NAc punches were homogenized in 500 μl of lysis buffer consisting of 25 mm HEPES, pH 7.4, 500 mm NaCl, 2 mm EDTA, 20 mm NaF, 1 mm PMSF, 0.1% NP-40 (v/v), 1 mm NaOV, 1× protease inhibitor cocktail Set 1 (Millipore), 1% Triton, and 0.5% deoxycholate. Three micrograms of DGL-α antibody (a gift from Dr. Ken Mackie, Indiana University, Bloomington), validated in previous studies (Katona et al., 2006; Keimpema et al., 2010), was incubated overnight at 4°C with protein A/G agarose slurry. The pellet containing antibody-coated beads was then incubated overnight at 4°C with 100 μg of NAc core tissue homogenate. The agarose-bound antibody was pelleted by centrifugation to isolate the bound fraction (two rounds of immunoprecipitation were performed to maximize recovery). The combined bound fraction was suspended in 2× Laemmli sample buffer with 1× XT Reducing Agent (Bio-Rad) in a volume equal to the input tissue volume. Samples were heated to 100°C for 3 min and stored at −20°C. Samples were run on 4–12% Bis-Tris gels (BIO-RAD) and transferred to PVDF membranes for immunoblotting. Membranes were then washed in distilled H_2_O (dH_2_O) and blocked with 1% normal goat serum with 5% nonfat dry milk in 0.05% Tween-20 in TBS, pH 7.4, for 1 h at room temperature. Membranes were incubated overnight at 4°C with antibodies to mGlu5 (1:10,000; catalog #AB5675, Millipore Sigma), CaMKII (1:1000; catalog #3362, Cell Signaling Technology), p-Thr286 CaMKII (1:1000; catalog #p1005-286, Phospho Solutions), p-Ser (1:120; catalog #sc-81 514, Santa Cruz Biotechnology), Homer1b/c (1:200; catalog #sc-55 463, Santa Cruz Biotechnology), or Homer2 (1:500; catalog #H00009455-B01P, Abnova). The mGlu5 dimer band (∼260 kDa) was analyzed because it represents the functional pool of these receptors (Jingami et al., 2003). GAPDH was used as a loading control when immunoblotting total tissue homogenates. Secondary antibodies to rabbit or mouse IgG light chain were used (catalog #211–032-171 or #115–035-174, Jackson ImmunoResearch) in co-IP studies to prevent interference with the Homer band (∼45 kDa) by the heavy chain (∼50 kDa). Visualization was achieved by chemiluminescence (GE Healthcare). Immunoblots were analyzed with TotalLab (Life Sciences Analysis Essentials). Data were excluded only if imperfections in the gel or blot interfered with analysis. Across all blots, data points for two samples were removed from analysis. These consisted of one cocaine and one saline data point from the synaptoneurosome preparation.

### Synaptoneurosome preparation

Rats were decapitated and bilateral NAc tissue (primarily core) was rapidly dissected from a 2 mm coronal slice prepared with a brain matrix (ASI Instruments). Immediately following dissection, synaptoneurosomes were prepared according to published protocols (Most et al., 2015; Workman et al., 2015; Werner et al., 2018). NAc punches were homogenized in 500 μl of homogenization buffer [HB; 20 mm HEPES, 0.5 mm EGTA, 1× Proteasome Inhibitor Cocktail Set 1 (Millipore)]. Homogenates were passed through a 100-μm-pore filter and then through a 5-μm-pore filter (Millipore; both filters were prewashed with HB). After homogenates were passed through each filter, filters were washed with 50 μl of HB, and the washes were added to homogenates to maximize yield. Homogenates were then centrifuged at 14,000 × *g* for 20 min at 4°C. The pellet, which contains the synaptoneurosomes, was frozen on dry ice, stored at −80°C, and ultimately lysed in lysis buffer [0.605 × *g* Tris-HCl, 0.25 × *g* sodium deoxycholate, 0.876 × *g* NaCl, 1 μg/ml PMSF, 5 ml of 20% SDS, and 1× Protease Inhibitor Cocktail Set 1 (Millipore) in 100 ml of dH_2_O] for immunoblotting. NAc synaptoneurosomes prepared from individual rats (10 μg protein/lane) were mixed 1:1 with 2× sample treatment buffer (catalog #161–0737, BIO-RAD) and analyzed by SDS-PAGE and immunoblotting. β-Tubulin was used as a loading control.

### DGL activity

Rats were decapitated, and bilateral NAc was rapidly dissected as described above for synaptoneurosome preparation. Lipase activity was subsequently assayed using fluorescence resonance energy transfer (FRET) as adapted from a previous report (Johnston et al., 2012). Briefly, 600 μl of homogenization buffer [50 mm HEPES, pH 7.0, 250 mm sucrose, 1 mm Roche PhosStop (Millipore Sigma), 0.5 mm tris (2-carboxyethyl)phosphine (TCEP), 0.01 mm leupeptin, 0.001 mm pepsin] was added to punched NAc tissue and macerated by a 27 gauge needle. The solution was homogenized with an electric pestle and then centrifuged for 30 min. Supernatant was removed from the pellet and discarded. The pellet was resuspended in 300 μl of membrane resuspension buffer (same as homogenization buffer but without sucrose), then vortexed and briefly sonicated on ice. Using a Bradford assay, protein concentration of the punched NAc tissue was determined. A 96-well plate was then prepared on ice with protein samples in triplicate at 40 μg total protein/well. As a negative control, tissue from cerebellum was run in the absence and presence of the DAGL inhibitor DO34 (2 μm). The FRET reporter compound MRJ20 (compound #17 in Johnston et al., 2012), a FRET-based substrate for DGL, was made in-house (Vanderbilt Chemical Synthesis Core, Nashville, TN). MRJ20 was added to the assay buffer (50 mm HEPES, pH 7.0, 0.5 mm TCEP, 1 mm PhosStop) to reach a final concentration of 2 μm. The assay buffer was then vortexed and sonicated on ice for 2 min. The plate was then taken to a prewarmed fluorescent plate reader (Synergy H4, BioTek), and the recently sonicated assay buffer was pipetted into the wells at 150 μl. The reader maintained a constant 37°C temperature and took fluorescent measurements every minute for 30 min. Samples were then analyzed for relative fluorescent units (RFU) over the 30 min, and a line of best fit for RFU per minute determined relative slopes (ΔRFU/minute) for each sample (GraphPad Prism).

### Statistical analyses

Data are expressed as the mean ± SEM. Groups were compared with unpaired (between group) or paired (within group) *t* tests. Pearson correlation coefficients were used to assess the linear correlation between the magnitude of incubation of craving and DGL activity. Differences between experimental groups were considered statistically significant when *p* < 0.05.

## Results

### Incubation of cocaine craving is not associated with changes in DGL expression or its association with mGlu5 receptors and Homer scaffolding proteins

**Figure 1.**
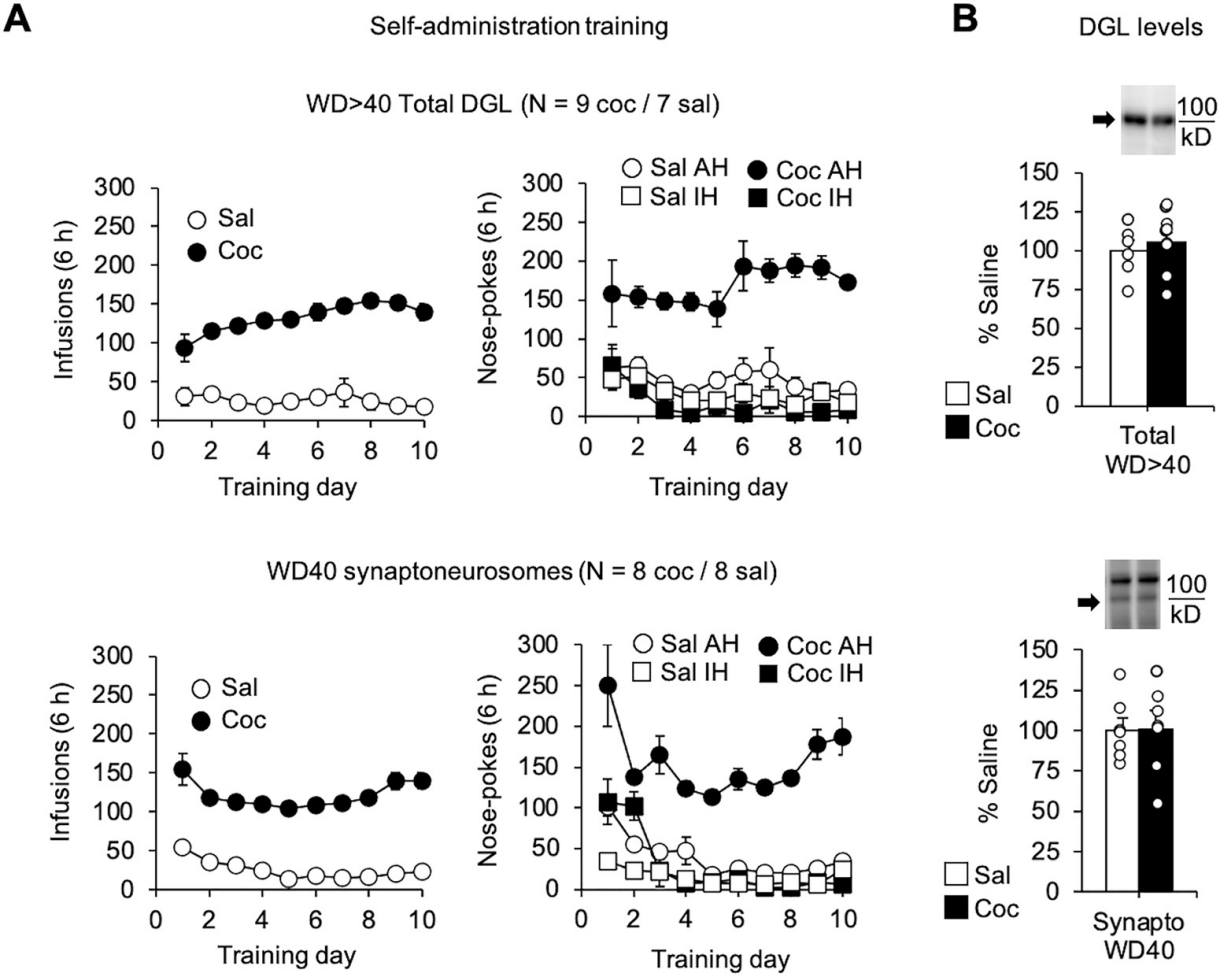
DGL protein levels in the NAc core are unchanged after prolonged withdrawal from extended-access cocaine (Coc) self-administration. ***A***, Two cohorts of rats underwent extended-access self-administration of cocaine or saline (6 h/d for 10 d), destined for preparation of NAc core homogenates after WD40 (WD > 40; top) or NAc synaptoneurosomes on WD40 (bottom). Shown are the number of infusions (left graphs) or number of active hole (AH) and inactive hole (IH) nose pokes (right graphs) on the 10 d of self-administration training. *N* values are given in the figure. ***B***, DGL protein levels do not differ between saline and cocaine groups based on immunoblot analysis of NAc homogenates (top) or synaptoneurosomes (bottom). Data are expressed as the percentage of controls (*n* = 7–9 rats/group; mean ± SEM). Representative lanes from immunoblots (cropped) are shown for a saline rat (left) and a cocaine rat (right). Arrows indicate bands analyzed, and lines indicate the location of the molecular weight marker. Saline and cocaine groups were compared with unpaired *t* tests (n.s.).

**Figure 2.**
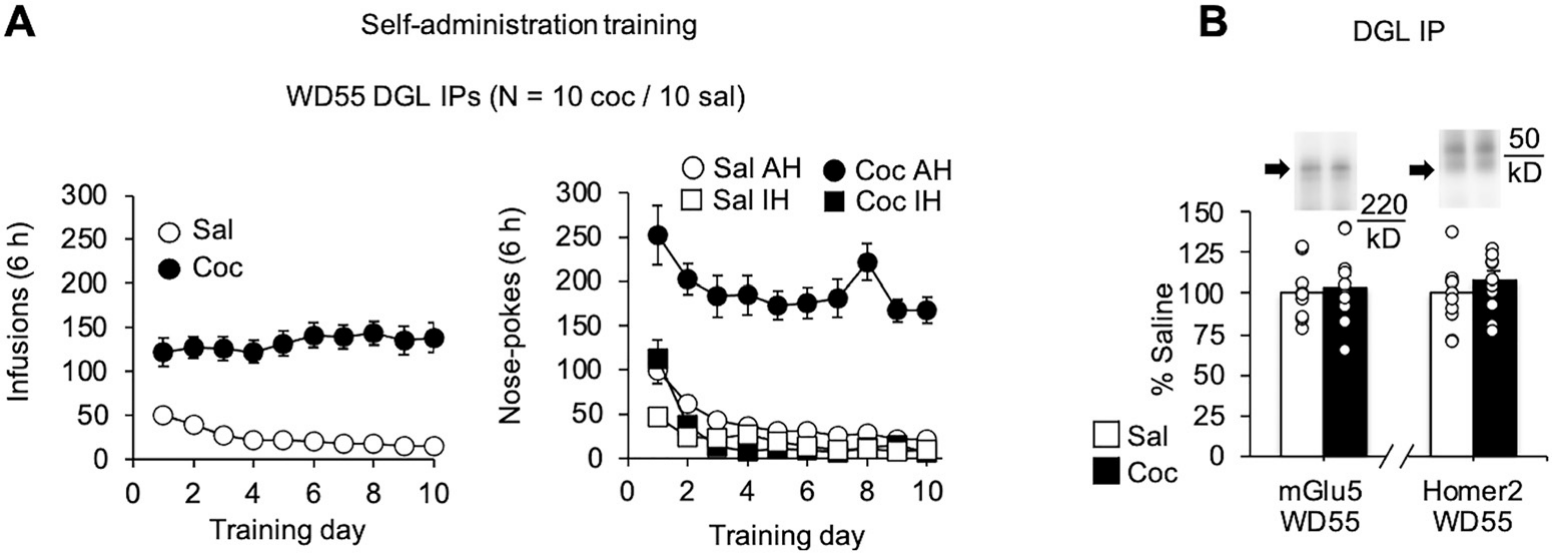
Associations between components of the 2-AG signalosome in the NAc core are unchanged after prolonged withdrawal from extended-access cocaine (Coc) self-administration. ***A***, Rats underwent extended-access self-administration of cocaine or saline (6 h/d for 10 d), destined for the preparation of NAc core homogenates on WD55. Shown are the number of infusions (left graph) or the number of active hole (AH) and inactive hole (IH) nose pokes (right graph) during the 10 d of self-administration training. *N* values are given in the figure. ***B***, Coimmunoprecipitation experiments assessing the physical associations between DGL and mGlu5 or Homer2 in NAc tissue obtained on WD55 (also see Extended Data Figure 2-1). DGL was immunoprecipitated from NAc core homogenates, and the bound fraction was immunoblotted for mGlu5 or Homer2. Data are expressed as the percentage of saline (Sal) controls (*n* = 9–10 rats/group; mean ± SEM). Representative lanes from immunoblots (cropped) are shown for a saline rat (left) and a cocaine rat (right). Arrows indicate bands analyzed, and lines indicate the location of the molecular weight marker. IB, Immunoblot; IP, Immunoprecipitation. Saline and cocaine groups were compared with unpaired *t* tests (n.s.).

Three cohorts of rats underwent extended-access saline or cocaine self-administration (10 sessions of 6 h/d; the three cohorts are depicted in Fig. 1*A* top, Fig. 1*A* bottom, and Fig. 2*A*). In all cases, biochemical analyses of NAc were conducted on or after WD40, when incubation of cocaine craving has plateaued (Lu et al., 2004) and stable CP-AMPAR elevation (Wolf and Tseng, 2012) and loss of mGlu5-dependent synaptic depression (McCutcheon et al., 2011; Scheyer et al., 2018) have been observed. The core subregion was analyzed, because of its critical role in the incubation of cocaine craving (Conrad et al., 2008; Guillem et al., 2014; Loweth et al., 2014). In NAc homogenates prepared from the first cohort of rats (Fig. 1*A*, top), we found that DGL levels did not differ between cocaine and saline groups (Fig. 1*B*, top). NAc tissue from the second cohort (Fig. 1*A*, bottom) was used to prepare synaptoneurosomes, a subcellular fraction enriched for the postsynaptic density (Hollingsworth et al., 1985; Quinlan et al., 1999; Most et al., 2015; Workman et al., 2015), on WD40. DGL levels in NAc synaptoneurosomes likewise did not differ between cocaine and saline groups (Fig. 1*B*, bottom).

NAc tissue from the third cohort was used to assess the DGL associations with Homer2 and mGlu5 by immunoprecipitating DGL and measuring bound Homer and mGlu5 levels (Fig. 2). mGlu5 and its association with Homer scaffolding proteins are necessary for 2-AG signaling at excitatory synapses (Roloff et al., 2010) and DGL associations with Homer scaffolding proteins are required for membrane-bound DGL activity (Jung et al., 2007). Although DGL does not directly bind to mGlu5, we detected bound mGlu5 in DGL immune complexes (Fig. 2*B*, Extended Data Fig. 2-1*A*) and also detected bound DGL in mGlu5 immune complexes (Extended Data Fig. 2-1*A*), presumably via intermediate Homer interactions. Homer1b/c was not detected in our DGL immune complexes in NAc core (Extended Data Fig. 2-1*B*,*C*). We therefore focused on Homer2, which has been implicated in the actions of cocaine in the NAc (Szumlinski et al., 2008). DGL associations with Homer2 and mGlu5 were unaffected by cocaine self-administration and prolonged withdrawal (Fig. 2*B*). These results suggest that impairments in physical association between these components of the 2-AG signalosome are not responsible for impaired mGlu5-dependent synaptic depression after incubation of cocaine craving.

10.1523/ENEURO.0220-21.2021.f2-1 Figure 2-1 Assessment of associations between DGL, mGlu5, and Homer1b/c in the NAc of drug-naive animals. ***A***, Coimmunoprecipitation experiments assessing the association between DGL and mGlu5. Left, Results of an experiment in which DGL was immunoprecipitated from NAc core homogenates, followed by immunoblotting for mGlu5 in the starting material (SM; homogenate), Bound (B) or immunoprecipitated fraction, and unbound (UB) fraction. mGlu5 is present in all fractions. Right, Results after immunoprecipitating mGlu5 from the same starting material and immunoblotting for DGL. Although DGL is more abundant in starting material and unbound fractions, it is detectable in the bound fraction. ***B***, ***C***, Coimmunoprecipitation experiments assessing the association between DGL and Homer1b/c. Although Homer1b/c is present in the starting material (***B***), it is not detected in the bound fraction after immunoprecipitation of DGL (***C***); the arrow in ***C*** shows the molecular weight at which the Homer1b/c band should have been observed. IB, Immunoblot. Download Figure 2-1, TIF file.

### Incubation of cocaine craving is associated with increased interactions between CaMKII and DGL

**Figure 3.**
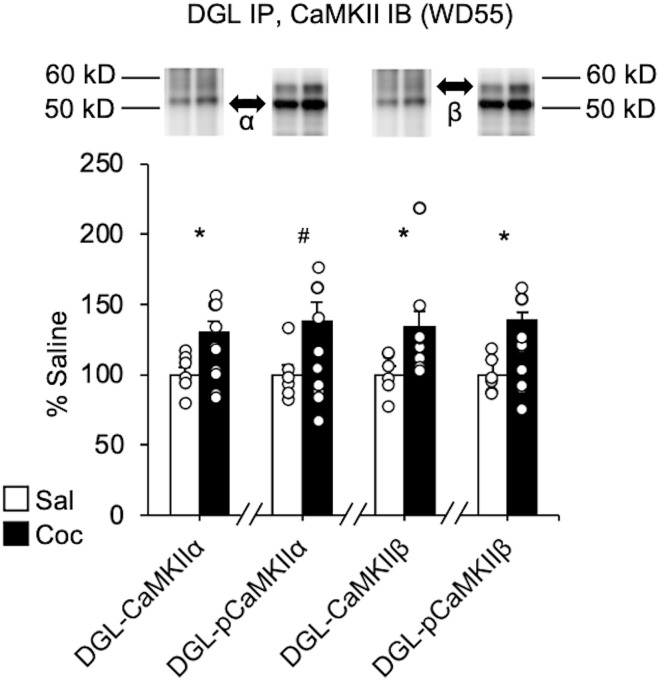
The association of CaMKII with DGL is increased in the NAc core after prolonged withdrawal from extended-access cocaine (Coc) self-administration. Coimmunoprecipitation experiments assessed the physical associations between DGL and phosphorylated or total CaMKIIα and CaMKIIβ in NAc core homogenates prepared on WD55 (same rats for which behavioral data are shown in Fig. 2). Also see Extended Data Fig. 3-1. Data are expressed as the percentage of the saline (Sal) control group (*n* = 6–10 rats/group; mean ± SEM; unpaired *t* tests, *p < 0.05, ^#^*p* = 0.0501 vs saline). Representative lanes from immunoblots (cropped) are shown for a saline rat (left) and a cocaine rat (right). Arrows indicate bands analyzed, and lines indicate the location of the molecular weight marker. IB, Immunoblot.

The loss of mGlu5-dependent synaptic depression after incubation of cocaine craving may be attributable to interactions that modulate DGL activity. For instance, CaMKIIα and CaMKIIβ bind DGL, and studies in dorsal striatum have found that CaMKII phosphorylation of DGL inhibits its activity and, conversely, that 2-AG-dependent depolarization-induced suppression of excitation is augmented by pharmacological inhibition of CaMKII (Shonesy et al., 2013). Furthermore, increased CaMKII activity is implicated in stress-induced impairment of endocannabinoid-mediated synaptic depression in the lateral habenula (Park et al., 2017). To determine whether interactions between DGL and CaMKII are affected during late withdrawal from cocaine self-administration, different aliquots of the same tissue samples used for the experiment depicted in Figure 2 were used to immunoprecipitate DGL and measure bound CaMKII by immunoblotting. DGL associations with total CaMKIIα (*t*_(14)_ = 2.59, *p *=* *0.021), total CaMKIIβ (*t*_(14)_ = 2.30, *p *=* *0.037), phosphorylated (Thr286) CaMKIIα (*t*_(14)_ = 2.14, *p *=* *0.050), and phosphorylated CaMKIIβ (*t*_(14)_ = 2.59, *p *=* *0.014) were all increased in the cocaine group relative to saline controls (Fig. 3). CaMKII phosphorylates DGL on two serine residues (Shonesy et al., 2013). Therefore, to determine whether CaMKII associations with DGL also led to detectable increases in DGL phosphorylation, other aliquots of these DGL immunoprecipitated samples were subjected to SDS-PAGE followed by immunoblotting with an antibody that recognizes phosphorylated serine residues. A band was detected at the molecular weight of DGL (∼100 kDa), but its intensity did not differ between saline and cocaine groups (Extended Data Fig. 3-1). However, these data are not conclusive. Whereas the lysis buffer for initial homogenization of tissue contained a reagent for inhibiting phosphatases (20 mm NaF), our washing buffer for processing the bound fraction during the immunoprecipitation protocol did not, because at the time the tissue was processed, we did not intend to use it to detect protein phosphorylation. Therefore, DGL might have been dephosphorylated during the lengthy immunoprecipitation protocol (see Materials and Methods).

10.1523/ENEURO.0220-21.2021.f3-1 Figure 3-1 Assessment of DGL phosphorylation in the NAc after prolonged withdrawal from extended-access cocaine (Coc) self-administration. Rats underwent extended-access self-administration of cocaine or saline (6 h/d for 10 d), destined for preparation of NAc core homogenates on WD55 (Fig. 2, behavioral data). DGL was immunoprecipitated from NAc core homogenates (six saline rats, nine cocaine rats), and the bound fraction was immunoblotted with an antibody that recognizes phosphorylated serine residues (p-Serine). No group difference was found (*t*_(13)_ = 2.54, *p* = 0.897). However, as noted in the main text, these data are not conclusive. Because we did not originally plan to assess the phosphorylation state of DGL using this tissue, some of the buffers used in the immunoprecipitation protocol did not contain phosphatase inhibitors. Thus, DGL may have been dephosphorylated during the lengthy immunoprecipitation protocol. IB, Immunoblot. Download Figure 3-1, TIF file.

### DGL activity is reduced in the NAc core after incubation of cocaine craving

**Figure 4.**
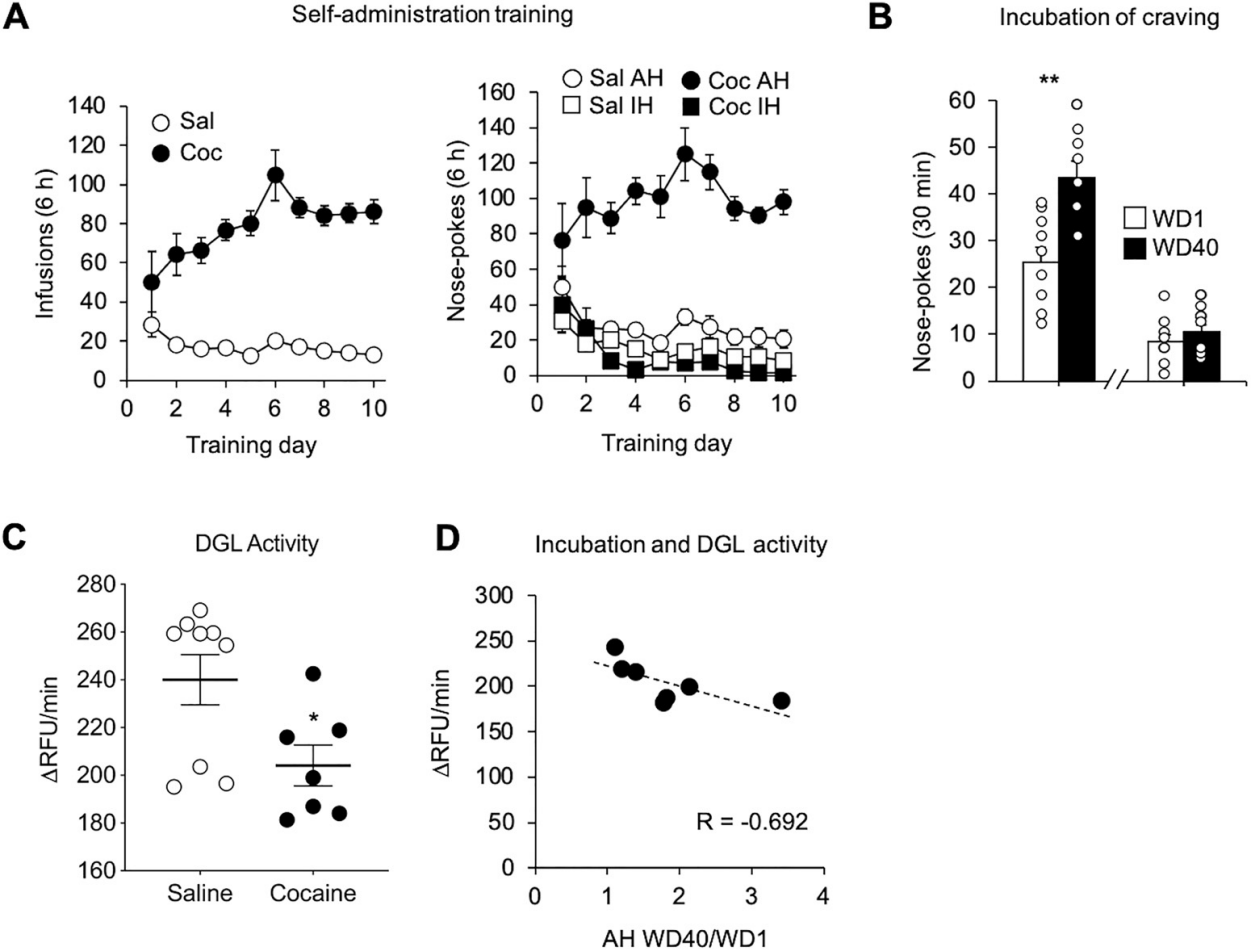
Reduction of DGL activity in the NAc after prolonged withdrawal from extended-access cocaine (Coc) self-administration. ***A***, Training. Rats self-administered saline or cocaine for 6 h/d for 10 d (*n* = 9 rats/group). Shown are the number of infusions (left graph) or number of active hole (AH) and inactive hole (IH) nose pokes (right graph). ***B***, Seeking tests on WD1 and WD40. During the 30 min test, AH nose pokes led to contingent presentation of the 20 s light cue previously paired with cocaine infusion, but no cocaine. Shown are AH and IH nose pokes during the seeking test (mean ± SEM; paired *t* test, **p < 0.01 vs WD1). ***C***, DGL activity. Five days after the WD40 seeking test, bilateral NAc punches comprised primarily of NAc core were snap frozen. Fluorescence resonance energy transfer assay of DGL activity was then conducted and results expressed as ΔRFU/minute. The cocaine group shows reduced DGL activity (*n* = 7–9 rats/group; mean ± SEM; unpaired *t* test, *p < 0.05 vs saline). ***D***, Linear regression evaluating the relationship between DGL activity in NAc core of cocaine animals versus the magnitude of incubation of craving expressed as WD40/WD1 AH nose pokes (*n* = 7 rats; *R* = −0.692, *p* = 0.085).

Based on earlier studies (Shonesy et al., 2013; Park et al., 2017), the observation of increased CaMKII association with DGL suggests that DGL activity will be reduced after prolonged withdrawal from extended-access cocaine self-administration. To test this, a new cohort of rats was generated (nine saline and nine cocaine; Fig. 4*A*). Cue-induced seeking tests were conducted on WD1 and WD40 to confirm incubation of craving (*t*_(16)_ = 3.748, *p *=* *0.002; Fig. 4*B*). On WD45, NAc tissue (primarily core) was harvested and snap frozen. DGL activity was subsequently assayed using FRET (Johnston et al., 2012). Compared with saline controls, the cocaine group was found to have reduced DGL activity (*t*_(14)_ = 2.54, *p *=* *0.024; Fig. 4*C*). Furthermore, in this initial cohort there was a trend for an inverse relationship between DGL activity in late withdrawal (WD40) and the magnitude of incubation of craving for rats in the cocaine group, expressed as the ratio of active hole nose pokes during 30 min seeking tests conducted on WD40 versus WD1 for each rat (*p *=* *0.08; Fig. 4*D*).

**Figure 5.**
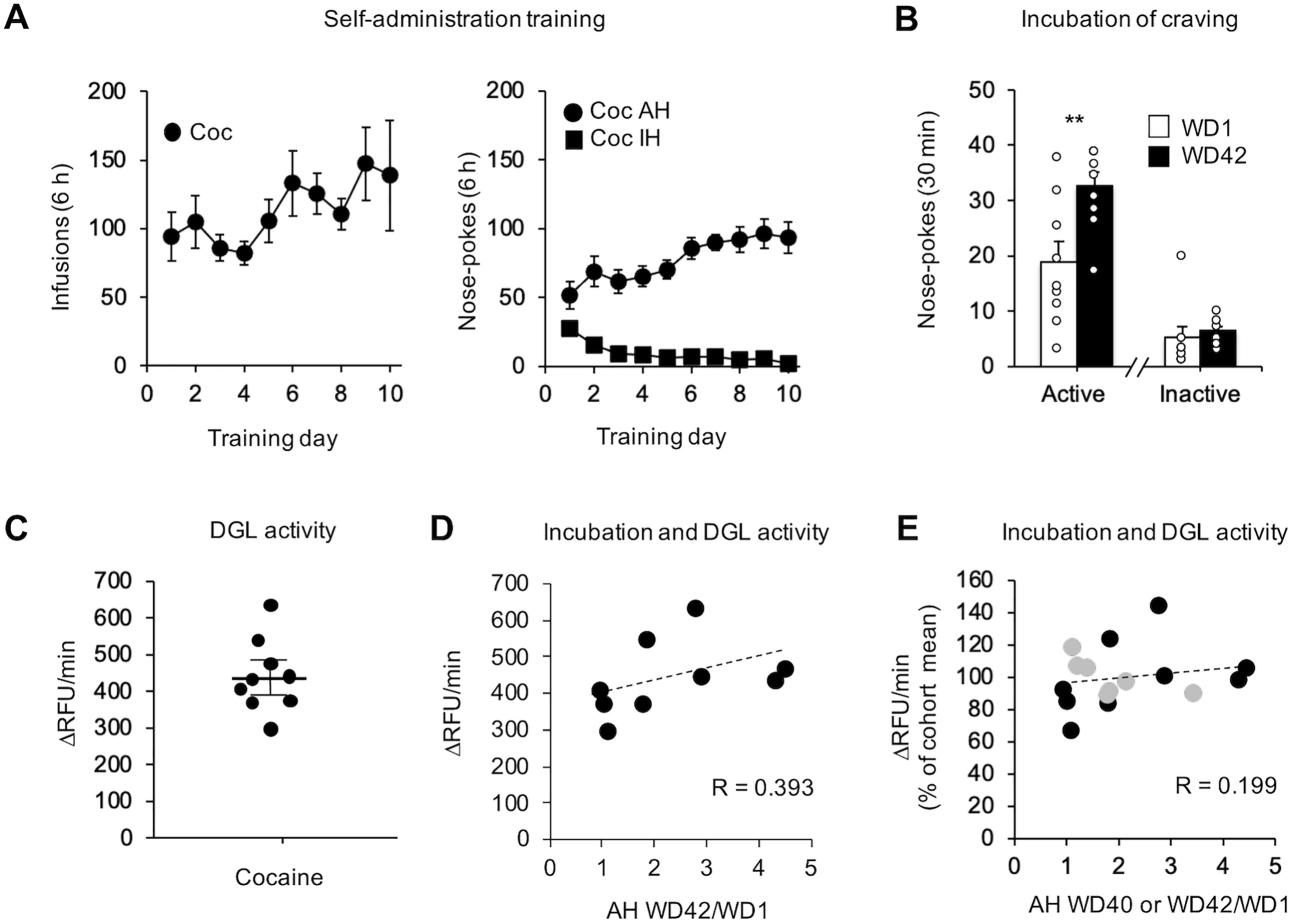
DGL activity in the NAc after prolonged withdrawal from extended-access cocaine (Coc) self-administration does not correlate with the magnitude of incubation of craving. ***A***, Training. Rats self-administered cocaine for 6 h/d for 10 d (*n* = 9 rats). Shown are the number of infusions (left graph), and the number of active hole (AH) and inactive hole (IH) nose pokes (right graph). ***B***, Seeking tests on WD1 and WD42. Shown are AH and IH nose pokes during the 30 min seeking test (mean ± SEM; paired *t* test, **p < 0.01 vs WD1). ***C***, Five days after the WD42 seeking test, bilateral NAc punches comprised primarily of NAc core were snap frozen and DGL activity was determined using fluorescent resonance energy transfer (Extended Data Fig. 5-1). ***D***, Linear regression evaluating DGL activity versus the magnitude of incubation of craving expressed as WD42/WD1 AH nose pokes. No significant correlation between DGL activity and incubation was found (*n* = 9 rats; *R* = 0.393; n.s.). ***E***, Combined linear regression evaluating DGL activity versus the magnitude of incubation of craving from both cocaine cohorts (shown in Fig. 4*A* and Fig. 5*A*). From comparison of Fig. 4*C* and Fig. 5*C*, it is apparent that absolute RFU per minute values for the two cohorts differed substantially (the cohorts were run over a year apart). To enable the data from the cohorts to be combined, mean values (ΔRFU/minute) were calculated for each cohort and individual rat values (ΔRFU/minute) were expressed as percentage of the cohort mean (Fig. 5*A* cohort in black, *n* = 7; Fig. 4*A* cohort in gray, *n* = 9; *R* = 0.199, n.s.). RFU, Relative Fluorescence Units.

We attempted to replicate this using an additional cohort of late withdrawal cocaine rats (Fig. 5*A*) for which incubation of craving was demonstrated (Fig. 5*B*). In this second cohort, we observed no significant relationship between DGL activity and the magnitude of incubation (Fig. 5*D*). We also assessed this relationship after combining the two cohorts. The second cohort was run over a year after the first, and required re-establishment of the assay. Perhaps for this reason, absolute RFU/minute values for the two cohorts differed substantially (compare Fig. 4*C* with 5*C* and Extended Data Fig. 5-1). Because the second cohort did not contain saline controls (they were not run because our focus was on understanding the correlation between DGL activity and incubation in the cocaine group), we could not combine the two cohorts by normalizing to saline control values. As an alternative approach, mean values (ΔRFU/minute) were calculated for each cohort, and individual rat values were expressed as a percentage of the cohort mean. We found no significant difference between DGL activity and incubation when the cohorts were combined in this manner (Fig. 5*E*). Overall, these results show that DGL activity is reduced in the NAc core after prolonged withdrawal from extended-access cocaine self-administration, and that the level of DGL activity in late withdrawal cocaine rats does not correlate with the magnitude of incubation.

10.1523/ENEURO.0220-21.2021.f5-1 Figure 5-1 DGL activity measurements in NAc core of cocaine rats and in two control groups. Rats self-administered cocaine (Fig. 5*A*) and received cue-induced seeking tests on WD1 and WD42 (Fig. 5*B*). Five days after the WD42 seeking test, bilateral NAc punches composed primarily of NAc core were snap frozen and DGL activity was determined using fluorescent resonance energy transfer. Triplicate values for each of nine cocaine rats are shown, along with duplicates for two control groups: cerebellum tissue (CB) and cerebellum tissue plus the DGL inhibitor DO34 (CB + DO34). Download Figure 5-1, TIF file.

## Discussion

Enduring vulnerability to relapse is encoded by synaptic plasticity in the NAc (Lüscher, 2016; Scofield et al., 2016; Wolf, 2016). An important form of synaptic depression in the NAc is elicited by postsynaptic mGlu5 stimulation, resulting in the generation of eCBs, which travel in a retrograde fashion to stimulate presynaptic CB1 receptors and reduce glutamate release (see Introduction). After the incubation of cocaine craving, this mGlu5/CB1R-dependent synaptic depression is lost in the NAc core (McCutcheon et al., 2011; Scheyer et al., 2014, 2018). This is accompanied by reduced surface mGlu5 and reduced mGlu5-Homer interactions (Loweth et al., 2014). Coupled with intact synaptic depression in the NAc of cocaine-incubated animals in response to direct CB1R activation (McCutcheon et al., 2011), these results suggest a postsynaptic locus for the loss of mGlu5/CB1R-dependent synaptic depression.

Here, we assessed the postsynaptic mGlu5 signaling complex that mediates the generation of 2-AG (the 2-AG signalosome; Piomelli, 2014) in the NAc core subregion. No changes were found in DGL protein levels or its physical association with mGlu5 and Homer proteins (Figs. 1, 2), indicating that the loss of mGlu5/CB1R-dependent synaptic depression does not reflect reductions in physical associations between these components of the 2AG-signalasome. However, we observed increased associations between DGL and CaMKII (Fig. 3), an interaction previously demonstrated to result in DGL phosphorylation and reduced DGL activity (Shonesy et al., 2013). For these biochemical studies (Figs. 1-3), we did not demonstrate incubation of craving in the rats used, but we made measurements at withdrawal times (WD ≥ 40) when incubation is reliably demonstrated after the same regimen (Conrad et al., 2008; Loweth et al., 2014; Figs. 4, 5). Next, we measured the enzymatic activity of DGL, the enzyme that catalyzes 2-AG production (Piomelli, 2014). As might be expected from our observation of increased DGL–CaMKII association and previous results mentioned above (Shonesy et al., 2013), we observed a decrease in DGL activity in NAc core tissue obtained from rats demonstrated to have undergone incubation of cocaine craving compared with saline controls (Fig. 4). Although we lack evidence for a causal relationship between the increased DGL–CaMKII association and the observed reduction in DGL activity, it is tempting to speculate that the increased DGL–CaMKII association may contribute to the loss of mGlu5/CB1R-dependent synaptic depression after prolonged cocaine withdrawal. Finally, we found that DGL activity in late withdrawal (WD40 or WD42) was not significantly correlated with the magnitude of incubation (expressed as the ratio of active hole nose pokes during seeking tests conducted on WD40 or WD42 vs WD1) for individual cocaine rats (Figs. 4, 5).

We note that there are several limitations to our study. First, we did not include cocaine WD1 rats or yoked cocaine controls in our biochemical studies. These groups would be necessary to establish that observed changes are specific to incubation and not merely the result of cocaine self-administration or cocaine exposure, respectively. A second limitation of our study is that the use of homogenates or synaptoneurosomes precludes the detection of potentially cell type-specific (D_1_ vs D_2_ MSNs) or input-specific adaptations in DGL activity. This could be one reason why we failed to identify a clear role for DGL adaptations in the incubation of craving. For example, one prominent incubation-related adaptation, synaptic insertion of CP-AMPARs, can occur on both D_1_ and D_2_ MSNs (Terrier et al., 2016; Wolf, 2016) but demonstrates input specificity (Lee et al., 2013; Ma et al., 2014; Pascoli et al., 2014; Terrier et al., 2016). A third limitation is that saline controls were not included in the experiment shown in Figure 5, as discussed extensively in Results. Finally, male rats were used for this study, in accordance with the NIH grant supporting this project. Given recent work showing sex differences in the incubation of cocaine craving (Kerstetter et al., 2008; Nicolas et al., 2019), future studies should explore similar questions in female rats.

### Role for CaMKII in loss of mGlu5/CB1R-dependent synaptic depression after incubation of cocaine craving

CaMKII is a Ca^2+^-activated enzyme that is best known for its critical role in early and late phases of long-term potentiation (LTP) but also participates in many other mechanisms regulating synaptic function (Lisman et al., 2002, 2012; Hell, 2014; Bayer and Schulman, 2019). There are four isoforms derived from four genes (α, β, γ, and δ), with the CaMKIIα and CaMKIIβ isoforms mainly expressed in the brain. The regulatory site of each isoform contains a phosphorylation site (Thr286 for CaMKIIα and Thr287 for CaMKIIβ) that can generate autonomous kinase activity (see reviews cited above).

We explored a role for CaMKII in the loss of mGlu5/CB1R-dependent synaptic depression after incubation of craving based on studies in dorsal striatum showing that CaMKIIα and CaMKIIβ bind DGL, CaMKII phosphorylation of DGL inhibits its activity, and, conversely, that 2-AG-dependent depolarization-induced suppression of excitation is augmented by pharmacological inhibition of CaMKII (Shonesy et al., 2013). Furthermore, increased CaMKII activity is implicated in stress-induced impairment of endocannabinoid-mediated synaptic depression in the lateral habenula (Park et al., 2017). Consistent with these prior findings, we found increased associations between DGL and both total and phosphorylated CaMKIIα and CaMKIIβ in NAc core tissue from cocaine rats (examined at an incubation-relevant late withdrawal time) versus saline controls. Furthermore, we observed a significant reduction in DGL activity in rats that had undergone incubation of craving compared with saline controls. These results suggest that CaMKII contributes to loss of mGlu5/CB1R-dependent synaptic depression after incubation. In theory, slice physiology experiments could directly test this relationship by determining whether acute CaMKII inhibition restores mGlu5/CB1R-dependent synaptic depression in NAc core MSNs from rats that have undergone incubation of cocaine craving. A practical concern is that commercially available inhibitors are not effective against the autophosphorylated enzyme, although new inhibitors are under development (Nassal et al., 2020).

Our findings are not the first to implicate CaMKII in neuroadaptations in the NAc after cocaine self-administration. For example, CaMKIIα, but not CaMKIIβ, mRNA levels increased in NAc shell after cocaine self-administration and in cocaine-yoked controls relative to saline self-administering animals, likely via histone H3 acetylation; the increase correlated with motivation for cocaine measured using a progressive ratio schedule, while knockdown of CaMKIIα in the NAc shell decreased motivation for cocaine (Wang et al., 2010). In another study, cocaine self-administration followed by extinction training did not alter CaMKII phosphorylation in NAc shell or core; however, cocaine-primed reinstatement was associated with an increase in NAc shell CaMKII phosphorylation, and intra-NAc shell CaMKII inhibition via KN-93 attenuated reinstatement (Anderson et al., 2008). During abstinence, increased CaMKIIα phosphorylation in the NAc was found 24 h after discontinuing short-access cocaine self-administration relative to yoked saline controls, but this was not observed in a yoked cocaine group, immediately after discontinuing self-administration, or after self-administration and 7 d of abstinence (Caffino et al., 2014). In an incubation of cocaine craving study in which rats self-administered saline or cocaine under extended-access conditions, increased ratios of phosphorylated to total CaMKIIα and CaMKIIβ were found in NAc postsynaptic density fractions from cocaine rats on WD45 versus cocaine rats on WD1 and saline rats (Ferrario et al., 2011). CaMKII is also implicated in responding to other drugs of abuse. For example, CaMKII activity in NAc shell is required for the enhanced amphetamine intake and sensitized locomotor responding observed in rats previously sensitized to noncontingent amphetamine (Loweth et al., 2008, 2010, 2013).

How might CaMKII become activated during abstinence? While classical studies identified CaMKII activation following acute stimulation in slices (i.e., in conjunction with LTP), CaMKII may also contribute to homeostatic plasticity (Chen et al., 2014; Hell, 2014). Most notably, in cultured hippocampal neurons, prolonged AMPAR blockade (24 h) increases CaMKIIβ activity, which in turn scales up synaptic levels of homomeric GluA1 receptors; when blockade is discontinued, a presynaptic component of homeostatic adaptation (increased release probability) is revealed in response to Ca^2+^ entry via CP-AMPARs and the resulting retrograde signaling (Thiagarajan et al., 2002, 2005; Groth et al., 2011). We have suggested that AMPAR upregulation during cocaine abstinence reflects a homeostatic scaling up of synaptic strength, triggered by relative inactivity during abstinence versus the period of cocaine exposure; while this may normalize synaptic transmission during abstinence, it has the unfortunate consequence of enhancing excitatory responses to drug-related cues when they are presented after abstinence (Boudreau and Wolf, 2005; Conrad et al., 2008; Sun and Wolf, 2009). Likewise, the observation that sustained food restriction upregulates reward responding through the upregulation of NAc CP-AMPARs has been hypothesized to represent a homeostatic response (Carr, 2020). The finding of increased CaMKII phosphorylation in NAc postsynaptic density fractions prepared after incubation of cocaine craving (Ferrario et al., 2011) combined with increased phosphorylation of the DGL-bound CaMKII pool in the present study suggests that CaMKII may play a role after cocaine withdrawal that to some extent parallels that observed after AMPAR blockade in hippocampal neurons (above). The reduction in DGL activity that is observed in parallel with CaMKII activation could be “collateral damage” or it could contribute to the homeostatic response by removing a braking effect on excitatory synaptic transmission. It is also possible that CaMKII activation after incubation of craving is functionally relevant to DGL regulation, but that different homeostatic cascades are involved in CP-AMPAR upregulation (Wang et al., 2018; Loweth et al., 2019).

### Relationship between mGlu5/CB1R-dependent synaptic depression and cocaine seeking

As DGL activity was reduced in rats that underwent incubation of cocaine craving compared with saline controls (above), we tested for a correlation between DGL activity and the magnitude of incubation of craving (expressed relative to baseline craving on withdrawal day 1) for individual cocaine rats in two separate cohorts. Because the expression of incubation requires excitatory synaptic transmission onto NAc core MSN (Wolf, 2016), and DGL activity leading to 2-AG formation leads to reduced glutamate transmission (i.e., synaptic depression), we hypothesized that cocaine rats with the lowest DGL activity might show the strongest incubation of craving. However, we failed to observe a significant correlation between DGL activity and cocaine seeking. This does not rule out a contribution of reduced DGL activity to incubation. It is possible that reduction of DGL activity below a threshold level plays a permissive role in the enhancement of cue-induced cocaine seeking.

However, a number of lines of evidence argue against this possibility. First, mGlu5/CB1R-dependent synaptic depression has been linked to the promotion of reward seeking in mice with constitutive knockdown of mGlu5 in D_1_ receptor-expressing MSNs. These mice do not express mGlu5-dependent synaptic depression or demonstrate cue-induced reinstatement of cocaine or saccharin seeking; however, after 2-AG elevation with monoacylglycerol lipase (MAGL) inhibition, reinstatement of saccharin seeking is restored (Novak et al., 2010; Bilbao et al., 2020). Furthermore, it was found that DGL and MAGL were dysregulated after prolonged abstinence (30 d) from cocaine self-administration, and that intra-NAc administration of a DGL inhibitor at this withdrawal time reduced cue-induced cocaine craving, while inhibition of MAGL (to increase 2-AG levels) had the opposite effect (Mitra et al., 2021). We note that DGL levels were increased at WD30 in this prior study (Mitra et al., 2021), in contrast to the lack of change reported here. This is most likely because of the fact that Mitra et al. (2021) measured DGL in NAc shell, whereas we analyzed NAc core; there were also significant differences in the cocaine self-administration regimen. While the work cited above focused on synaptic depression expressed presynaptically via CB1R stimulation, other studies link mGlu5-dependent reinstatement of cocaine seeking to postsynaptically expressed LTD mediated via AMPAR internalization (Schmidt et al., 2013; Benneyworth et al., 2019).

Additional evidence against a causal relationship between loss of mGlu5/CB1R-dependent synaptic depression and elevation of cue-induced drug craving comes from studies on the incubation of methamphetamine craving. Dihydroxyphenylglycine-induced, mGlu5-dependent synaptic depression in the NAc core was lost during the first week of abstinence from extended-access methamphetamine self-administration, which corresponds to the rising phase of incubation of methamphetamine craving, but at later withdrawal times this synaptic depression was restored whereas craving remained at high “incubated” levels (Murray et al., 2021). These results argue against a role for mGlu5/CB1R-dependent synaptic depression in maintaining incubated craving for methamphetamine. It is also interesting to note that associations between CaMKII and DGL were unaltered during the first week of methamphetamine withdrawal when mGlu5/CB1R-dependent synaptic depression was lost (Murray et al., 2021), in contrast to the present results with cocaine. This adds to mechanistic distinctions between incubation of cocaine and methamphetamine craving, although commonalities also exist (Murray et al., 2019).

Overall, these results argue that synaptic depression can promote (Novak et al., 2010; Bilbao et al., 2020; Mitra et al., 2021) or be dissociated from (Murray et al., 2021) drug or natural reward seeking. Thus, the functional significance of the observed reduction in DGL activity observed after prolonged cocaine withdrawal remains unclear. Likewise, no consensus has emerged from work that has tested the effect of manipulation of eCB signaling on seeking for cocaine and other drugs of abuse. While reducing endocannabinoid signaling through negative allosteric modulation has shown therapeutic promise, so too has enhancement of endocannabinoid signaling through the inhibition of endocannabinoid degradation (Galaj and Xi, 2019). The complex nature of effects observed after pharmacological targeting of CB1Rs is likely to reflect their widespread expression (e.g., they are expressed not only on glutamate terminals but also on GABA terminals throughout the brain (Augustin and Lovinger, 2018). Furthermore, within the NAc, in addition to mediating synaptic depression onto MSNs, endocannabinoids also mediate synaptic depression onto parvalbumin-containing interneurons that regulate MSN activity (Manz et al., 2020) and contribute to the modulation of dopamine release (Mateo et al., 2017).

### Conclusion

The loss of mGlu5/CB1R-dependent synaptic depression in the NAc core after incubation of cocaine craving is likely attributable to multiple postsynaptic mechanisms, including reduced surface expression of mGlu5 and reduced mGlu5–Homer interactions (Loweth et al., 2014) and, as shown here, a reduction in DGL activity that may result from increased CaMKII–DGL association. Although our findings and related work in the literature suggest that the loss of mGlu5/CB1R-dependent synaptic transmission is unlikely to be a driver of the incubation of cocaine craving, it is expected that loss of a major form of synaptic plasticity would have significant consequences for NAc function. Future studies should continue to explore this problem.

## Synthesis

Reviewing Editor: Leandro Vendruscolo, The Scripps Research Institute Decisions are customarily a result of the Reviewing Editor and the peer reviewers coming together and discussing their recommendations until a consensus is reached. When revisions are invited, a fact-based synthesis statement explaining their decision and outlining what is needed to prepare a revision will be listed below. The following reviewer(s) agreed to reveal their identity: Brandon Warren.

Both reviewers expressed concerns about the lack of data for withdrawal day 1 to compare with later time points to indicate incubation in some experiments. Without these data, the authors may need to rephrase their statements to indicate that some of the biochemistry/molecular analyses were conducted at "incubation-relevant time point." They also expressed concerns about correlative findings without testing for causal role. However, I think that changes to the text will be enough to address their concerns without additional experiments.

Reviewer 1:

In the present study, the authors use an animal model of incubated cocaine craving to assess changes in diacylglycerol lipase-a (DGL) expression levels, enzymatic activity and associations with 2-AG signalosome in rat nucleus accumbens core followed extended access cocaine self-administration and >40 days of withdrawal. A key limitation of this study is that the authors do not assess these same effects immediately after self-administration (ie during early withdrawal) to show that the changes observed might be related to the incubation phenomenon. In the absence of this data one can only suggest that the effects observed are a result of cocaine self-administration and an extended period of withdrawal. Further, the results presented here are all correlational, and the conclusions are somewhat speculative in the absence of causal role experiments. This and other experimental issues listed below reduce my enthusiasm for this manuscript.

In experiment 1, the authors measured DGL expression and association with mGlu5 or Homer2 using NAc core tissue (homogenate or synaptoneurosome fractions) and found no differences between saline and cocaine groups following withdrawal. Based on this experiment the authors suggest that 2-AG signaling is not affected. However, no evidence is presented to support this claim. This data also disagrees with a similar study (PMID 33496035) that showed increases in DGL at WD30 but the authors do not discuss what could explain their results.

In experiment 2, the authors investigated changes in interaction between CaMKII isoforms and DGL at WD55 using synaptoneurosome fractions and find that both total and phosphorylated fractions of alpha and beta isoforms show increased interaction with DGL (compared to saline controls). While they discuss the possibility that this interaction could cause DGL phosphorylation (to explain the activity reduction on experiment 3) they again do not show any evidence to support this hypothesis. In the absence such causal experiments the two correlational findings should not be linked together. Minor point: The authors should check the axes in Fig. 2 as the error bar heights do not match the variability in individual data shown in the plots - 2 datapoints seem to be off scale in the cocaine-DGL-CaMKIIb group.

In experiment 3, the authors measure DGL activity at WD>40 and find reduced activity in the cocaine group (vs. saline). The hypothesize that this reduction is relevant to incubated responding and test for correlations between DGL activity and the 'degree' of incubation (measured as a ratio of WD40 responding over WD1 responding). The authors find opposite correlation patterns between two independent replications of the same experiment - in fact the range of values for delta-RFU is significantly different in the two replicates and the raw data for the second round is not shown. Overall, it seems incorrect to infer a role (or absence of one) in the development of incubated responding as no DGL activity measurements were made at WD1 to show that there was a change between WD1 and WD40. In the absence of this data one can only suggest that DGL activity is decreased following cocaine self-administration and an extended period of withdrawal. Further, there is no attempt to provide a functional link between DGL activity decrease and seeking - for example would administration of a DGL inhibitor potentiate seeking behavior in this task at WD55?

Reviewer 2

The present paper tests whether DGL activity changes after prolonged abstinence following cocaine self-administration. They first measure total DGL levels in the nucleus accumbens after 40 days of abstinence and find no differences between cocaine or saline self-administering rats. They next test whether DGL interacts with CaMKII using a co-immunoprecipitation assay and find that DGL and CaMKII coprecipitate more in cocaine self-administering than saline self-administering rats after prolonged abstinence. Lastly, they use FRET to test the activity of DGL-a after incubation of cocaine craving, but find no correlation between DGL-a activity and incubated cocaine seeking. Overall the paper is well-written and their conclusions are supported by their findings. My concerns are included below:

1. The paper would benefit from some reorganization. Figure 1 should be restructured so that it is clear which molecular data comes from which behavioral cohort. I suggest either breaking this into separate figures for each experiment, or at least restructuring the figure in such a way that the behavioral data and molecular data are linked. While these data were not significant, their presentation is currently somewhat confusing.

2. Did the authors assess incubation of cocaine craving in the rats trained in Figure 1? If so, these data should be included. If not, the authors should provide a brief justification for not measuring cocaine-seeking since it is unclear whether these rats actually had incubated cocaine craving.

3. Student's t-test is inappropriate for the data shown in fig 3B and 4B. These data should be analyzed using paired t-tests, since they are within subjects comparisons.

4. The authors state that some data was excluded from their analysis due to poor transfer. The number of excluded data points should be stated explicitly.

5. The authors indicate several pieces of data are not shown. These should be included as extended data to better support their statements.

6. The lack of yoked controls is problematic for interpretation of their DGL activity results. Their findings in Figure 3C could just represent long-lived cocaine-induced changes that are not tied to craving- although the correlation data suggest this is not the case. The authors should discuss this limitation.

7. Another potential limitation is the use of homogenate tissue. The NAc core is made up of multiple cell types (D1 and D2 MSNs in particular) that play differing, or even opposing, roles in incubation of cocaine craving. The authors should discuss briefly the possibility that DGL activity could differ between these cell types. This could be one reason that the authors do not find a clear role for this mechanism.

8. Is the DGL-a antibody a commercial antibody, or is it produced by Dr. Mackie? If it is commercial, please include the relevant source, and if it is produced, please provide a source validating the antibody in the methods.

9. Include the overall DGL activity figure (shown in figure 3C) for the replication shown in figure 4.

## Author Response

Authors Rebuttal eN-NWR-0220-21

Both reviewers expressed concerns about the lack of data for withdrawal day 1 to compare with later time points to indicate incubation in some experiments. Without these data, the authors may need to rephrase their statements to indicate that some of the biochemistry/molecular analyses were conducted at “incubation-relevant time point.” They also expressed concerns about correlative findings without testing for causal role. However, I think that changes to the text will be enough to address their concerns without additional experiments.

As detailed below, we have substantially revised the figures and text, including clarifying that we did not demonstrate incubation of craving for some rats and using the term “incubation-relevant time point” (or otherwise modifying our language to avoid implying that incubation was demonstrated) when describing results from these rats.

Reviewer 1:

In experiment 1, the authors measured DGL expression and association with mGlu5 or Homer2 using NAc core tissue (homogenate or synaptoneurosome fractions) and found no differences between saline and cocaine groups following withdrawal. Based on this experiment the authors suggest that 2-AG signaling is not affected. However, no evidence is presented to support this claim. We apologize for the misunderstanding. We did not intend to suggest that 2-AG signaling is unaffected (indeed we report reduced DGL activity in a subsequent experiment). Rather, our conclusion is that physical associations of these components of the 2-AG signalosome are not affected. To clarify this, we have revised the text (p. 10).

This data also disagrees with a similar study (PMID 33496035) that showed increases in DGL at WD30 but the authors do not discuss what could explain their results. We have revised the Discussion (p. 18) to state that this study (Mitra et al., 2021) found increased DGL on WD30 and to provide two possible explanations for the discrepancy: 1) they analyzed shell whereas we analyzed core (there are some significant differences in cocaine-induced neuroadaptations between these subregions), and 2) their cocaine self-administration regimen differed significantly from our regimen (which can also affect neuroadaptations).

In experiment 2, the authors investigated changes in interaction between CaMKII isoforms and DGL at WD55 using synaptoneurosome fractions and find that both total and phosphorylated fractions of alpha and beta isoforms show increased interaction with DGL (compared to saline controls). While they discuss the possibility that this interaction could cause DGL phosphorylation (to explain the activity reduction on experiment 3) they again do not show any evidence to support this hypothesis. In the absence such causal experiments the two correlational findings should not be linked together. This point is well-taken. We have revised the text (p. 13-14) to acknowledge lack of causal evidence and to be more cautious in our interpretation of these studies (also see last paragraph of Discussion). Minor point: The authors should check the axes in Fig. 2 as the error bar heights do not match the variability in individual data shown in the plots - 2 datapoints seem to be off scale in the cocaine-DGL-CaMKIIb group. We thank the reviewer for finding this graphical error; one individual datapoint was missing and is now included in the figure.

In experiment 3, the authors measure DGL activity at WD>40 and find reduced activity in the cocaine group (vs. saline). The hypothesize that this reduction is relevant to incubated responding and test for correlations between DGL activity and the ‘degree’ of incubation (measured as a ratio of WD40 responding over WD1 responding). The authors find opposite correlation patterns between two independent replications of the same experiment - in fact the range of values for delta-RFU is significantly different in the two replicates and the raw data for the second round is not shown. Overall, it seems incorrect to infer a role (or absence of one) in the development of incubated responding as no DGL activity measurements were made at WD1 to show that there was a change between WD1 and WD40. In the absence of this data one can only suggest that DGL activity is decreased following cocaine self-administration and an extended period of withdrawal. Many important points are raised here, which we will address in turn. First, we did not find “opposite correlation patterns” in the two independent cohorts, as illustrated when these data are plotted together in Fig. 5E. Rather there was a trend towards a negative correlation in the first cohort (Fig. 4D), and no trend in the second (Fig. 5D). To further clarify, we have replotted data in Fig. 4D to start from zero on the y axis, consistent with Fig. 5D. Second, the reason that the “range of values for delta-RFU is significantly different in the two replicates” is now stated in the legend to Fig. 5. The replicates/cohorts were run more than a year apart. After the first cohort was run, the person doing the work left the lab. When the second cohort was ready, the assay had to be re-established. Third, we have added a panel (Fig. 5C) showing raw data for the second cohort. Fourth, we have added clear acknowledgement that our study is limited by lack of WD1 cocaine data (p. 14). Fifth, we have revised our conclusion to correspond with the Reviewer’s suggestion above, although we think it is appropriate to mention lack of correlation between DGL activity in late withdrawal and the magnitude of incubation (WD1 vs WD40 or WD42) in the same rats (Revised text on p. 12: “Overall, these results show that DGL activity is reduced in the NAc core after prolonged withdrawal from extended-access cocaine self-administration, and that the level of DGL activity in late withdrawal cocaine rats does not corelate with the magnitude of incubation.”) We have similarly changed wording to refer to “prolonged withdrawal from extended-access cocaine self-administration” rather than “incubation of craving” on p. 12.

Further, there is no attempt to provide a functional link between DGL activity decrease and seeking - for example would administration of a DGL inhibitor potentiate seeking behavior in this task at WD55? Although we did not attempt to demonstrate a functional link, we noted in our original Discussion (now p. 19) that the interpretation of results from such experiments involves inherent limitations, given the various mechanisms involving 2-AG within the NAc beyond the mGlu5-mediated synaptic depression we would wish to target.

Reviewer 2

1. The paper would benefit from some reorganization. Figure 1 should be restructured so that it is clear which molecular data comes from which behavioral cohort. I suggest either breaking this into separate figures for each experiment, or at least restructuring the figure in such a way that the behavioral data and molecular data are linked. While these data were not significant, their presentation is currently somewhat confusing. Thank you for this suggestion. We have restructured Figure 1 and moved some behavioral data into Fig. 2 so that behavioral and molecular data from the same animals are always presented together. We have also revised the text to further clarify which rats were used for which molecular experiments (p. 9-10).

2. Did the authors assess incubation of cocaine craving in the rats trained in Figure 1? If so, these data should be included. If not, the authors should provide a brief justification for not measuring cocaine-seeking since it is unclear whether these rats actually had incubated cocaine craving. The rats in Figure 1 were not assessed for incubation of cocaine craving. We have revised the Methods section to clarify this and point out that other rats in this study, and past studies, that underwent the same cocaine self-administration regimen were verified to show incubation of craving (p. 6). We have also revised the Discussion (p. 13) to clarify that we did not demonstrate incubation in rats used for Figs 1-3.

3. Student’s t-test is inappropriate for the data shown in fig 3B and 4B. These data should be analyzed using paired t-tests, since they are within subjects comparisons. Thank you for catching this error. The data were analyzed with paired t-tests and we have revised the corresponding figure legends (now Figures 4 and 5) to indicate this.

4. The authors state that some data was excluded from their analysis due to poor transfer. The number of excluded data points should be stated explicitly. We have revised the Methods (p. 7) to specify that two data points were excluded.

5. The authors indicate several pieces of data are not shown. These should be included as extended data to better support their statements. We have created an Extended Data section that contains these pieces of data. Graphs from Extended Data are pasted below for convenience (see their legends in the Extended Data section for more detail). Here are the sentences from the main text that were revised to eliminate reference to “data not shown”:

Page 10: Although DGL does not directly bind to mGlu5, we detected bound mGlu5 in DGL immune complexes (Fig. 2B and Extended Data Fig. 2-1A) and also detected bound DGL in mGlu5 immune complexes (Extended Data Fig. 2-1A), presumably via intermediate Homer interactions. Homer1b/c was not detected in our DGL immune complexes in NAc core (Extended Data Fig. 2-1B,C).

Page 11: A band was detected at the molecular weight of DGL (∼100 kDa) but its intensity did not differ between saline and cocaine groups (Extended Data Fig. 3-1).

6. The lack of yoked controls is problematic for interpretation of their DGL activity results. Their findings in Figure 3C could just represent long-lived cocaine-induced changes that are not tied to craving- although the correlation data suggest this is not the case. The authors should discuss this limitation. We have revised the Discussion to acknowledge limitations in our ability to infer a role of DGL activity in the incubation of cocaine craving in the absence of cocaine WD1 or yoked cocaine data (p. 14). One consideration, which we note here but did not include in the revised text, is that yoked cocaine delivery is aversive (R.C. Twining et al., Behavioral Neuroscience 2009, PMC3861880), which would complicate interpretation of data from a yoked group.

7. Another potential limitation is the use of homogenate tissue. The NAc core is made up of multiple cell types (D1 and D2 MSNs in particular) that play differing, or even opposing, roles in incubation of cocaine craving. The authors should discuss briefly the possibility that DGL activity could differ between these cell types. This could be one reason that the authors do not find a clear role for this mechanism. The Reviewer identifies an important concern and we have added the suggested discussion (p. 14): “A second limitation of our study is that the use of homogenates or synaptoneurosomes precludes detection of potentially cell type-specific (D1 vs D2 MSN) or input-specific adaptations in DGL activity. This could be one reason why we failed to identify a clear role for DGL adaptations in the incubation of craving. For example, one prominent incubation-related adaptation, synaptic insertion of GluA2-lacking AMPA receptors, can occur on both D1 and D2 MSNs (Terrier et al., 2016; Wolf, 2016) but demonstrates input specificity (Lee et al., 2013; Ma et al., 2014; Pascoli et al., 2014; Terrier et al., 2016).” Along the same lines, both the original manuscript and the revised manuscript (p. 18) point out that our results do “not rule out a contribution of DGL activity to incubation.”

8. Is the DGL-a antibody a commercial antibody, or is it produced by Dr. Mackie? If it is commercial, please include the relevant source, and if it is produced, please provide a source validating the antibody in the methods. The antibody is produced by Dr. Mackie. We have cited two sources validating the antibody in the revised Methods section (p. 6).

9. Include the overall DGL activity figure (shown in figure 3C) for the replication shown in figure 4. If the reviewer is asking for a saline vs. cocaine figure, we did not include a saline group in the follow-up experiment because its purpose was to further explore the relationship between incubation of craving and DGL activity in late withdrawal cocaine rats. However, in response to this concern, we have included a new panel in Figure 5 (Figure 4 in the original submission) that shows individual data (in triplicate) for each of the cocaine rats as well as two control groups (panel C).
