# Supplementary figures and images for: CaMKII Modulates Diacylglycerol Lipase-α Activity in the Rat Nucleus Accumbens after Incubation of Cocaine Craving
Source: eNeuro. 2021 Oct 8;8(5):ENEURO.0220-21.2021. doi: 10.1523/ENEURO.0220-21.2021 (PMC8503962; doi:10.1523/ENEURO.0220-21.2021)

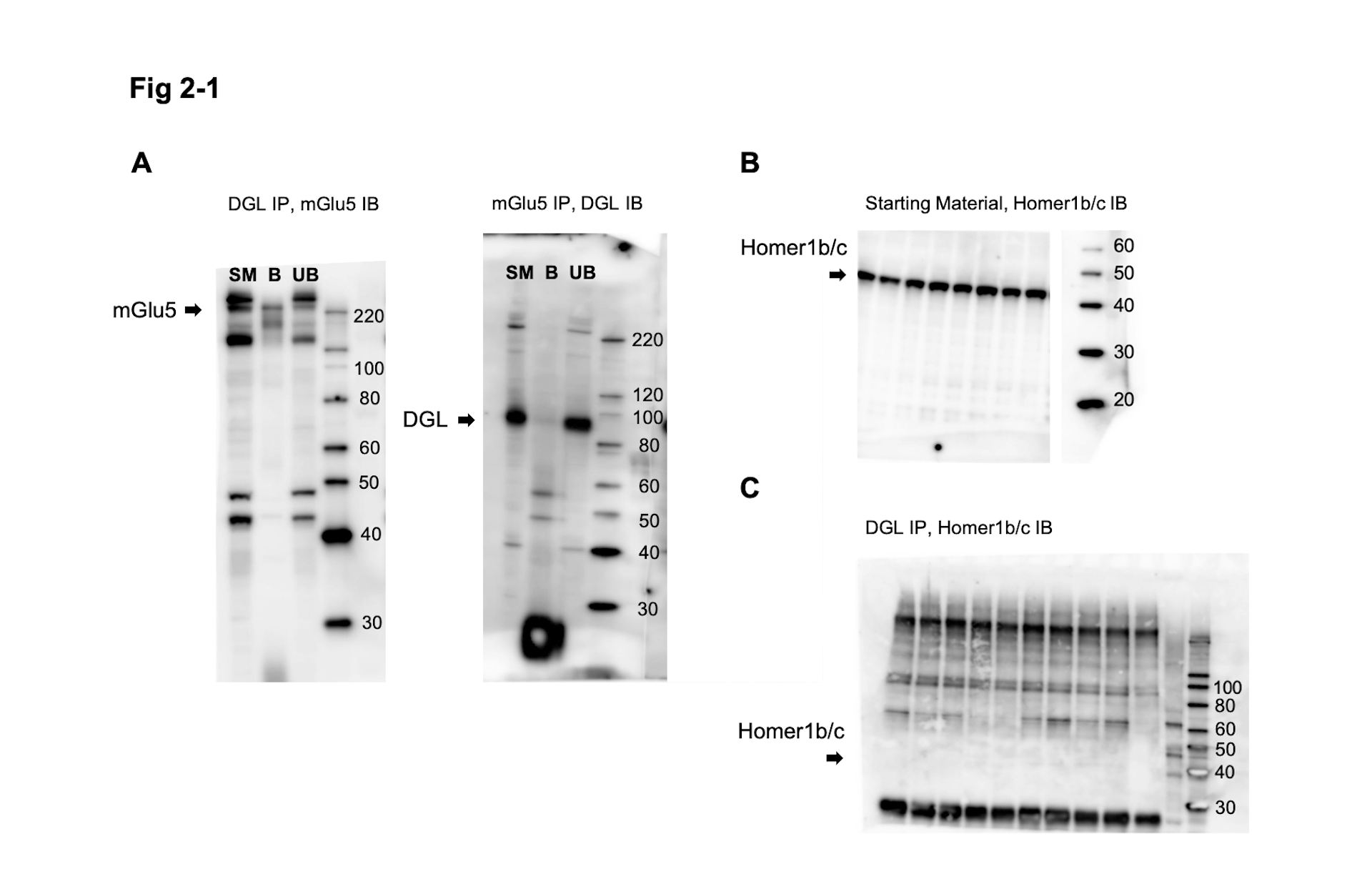

Supplement: Figure 2-1 — Assessment of associations between DGL, mGlu5, and Homer1b/c in the NAc of drug-naive animals. A, Coimmunoprecipitation experiments assessing the association between DGL and mGlu5. Left, Results of an experiment in which DGL was immunoprecipitated from NAc core homogenates, followed by immunoblotting for mGlu5 in the starting material (SM; homogenate), Bound (B) or immunoprecipitated fraction, and unbound (UB) fraction. mGlu5 is present in all fractions. Right, Results after immunoprecipitating mGlu5 from the same starting material and immunoblotting for DGL. Although DGL is more abundant in starting material and unbound fractions, it is detectable in the bound fraction. B, C, Coimmunoprecipitation experiments assessing the association between DGL and Homer1b/c. Although Homer1b/c is present in the starting material (B), it is not detected in the bound fraction after immunoprecipitation of DGL (C); the arrow in C shows the molecular weight at which the Homer1b/c band should have been observed. IB, Immunoblot. Download Figure 2-1, TIF file. [file enu-eN-NWR-0220-21-s01.tif]

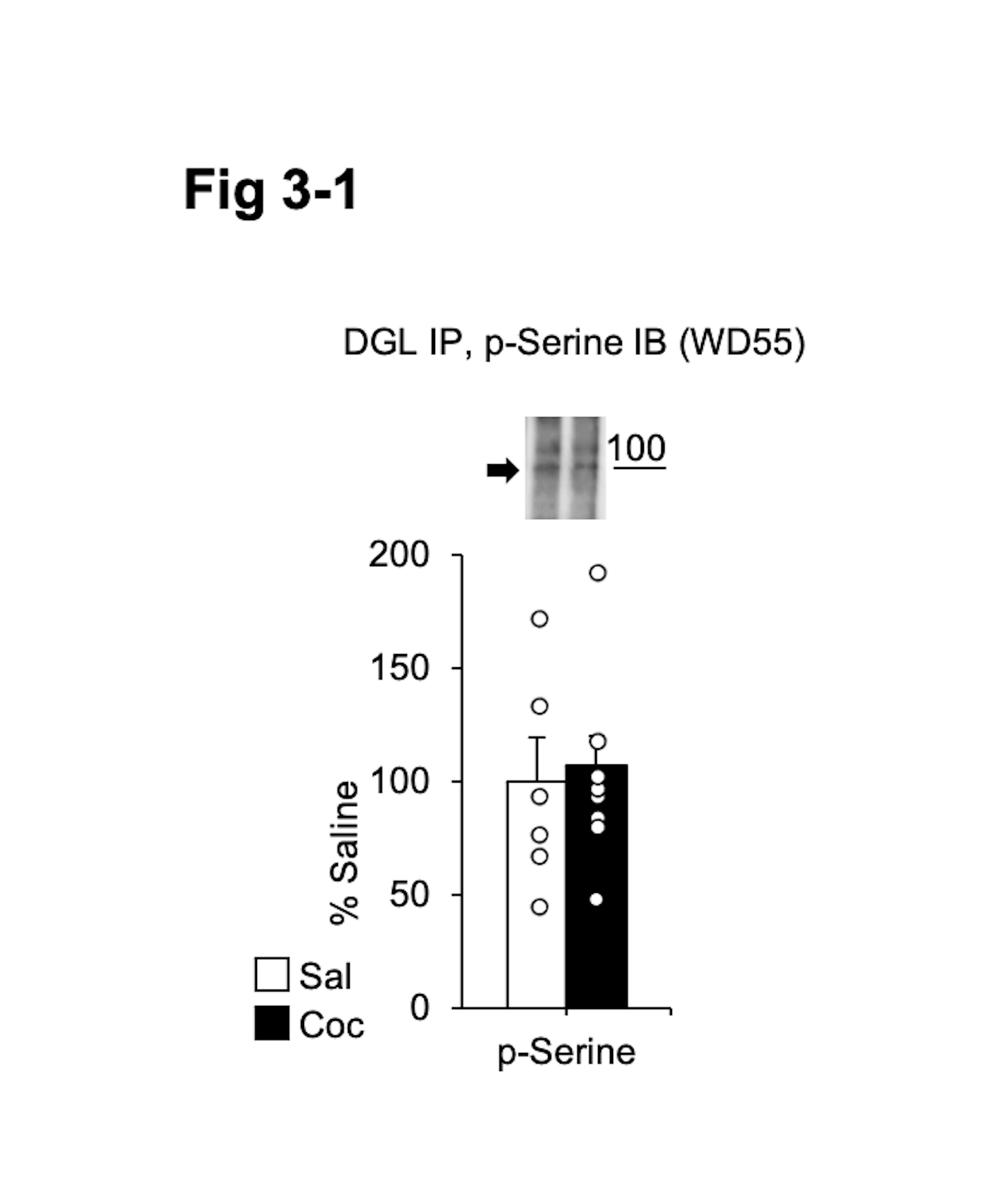

Supplement: Figure 3-1 — Assessment of DGL phosphorylation in the NAc after prolonged withdrawal from extended-access cocaine (Coc) self-administration. Rats underwent extended-access self-administration of cocaine or saline (6 h/d for 10 d), destined for preparation of NAc core homogenates on WD55 (Fig. 2, behavioral data). DGL was immunoprecipitated from NAc core homogenates (six saline rats, nine cocaine rats), and the bound fraction was immunoblotted with an antibody that recognizes phosphorylated serine residues (p-Serine). No group difference was found (t(13) = 2.54, p = 0.897). However, as noted in the main text, these data are not conclusive. Because we did not originally plan to assess the phosphorylation state of DGL using this tissue, some of the buffers used in the immunoprecipitation protocol did not contain phosphatase inhibitors. Thus, DGL may have been dephosphorylated during the lengthy immunoprecipitation protocol. IB, Immunoblot. Download Figure 3-1, TIF file. [file enu-eN-NWR-0220-21-s02.tif]
